# Optimizing cardiopulmonary rehabilitation duration for long COVID patients: an exercise physiology monitoring approach

**DOI:** 10.1007/s11357-024-01179-z

**Published:** 2024-05-21

**Authors:** Zsofia Szarvas, Monika Fekete, Gergo Jozsef Szollosi, Katica Kup, Rita Horvath, Maya Shimizu, Fuko Tsuhiya, Ha Eun Choi, Huang-Tzu Wu, Vince Fazekas-Pongor, Kinga Nedda Pete, Renata Cserjesi, Regina Bakos, Orsolya Gobel, Kata Gyongyosi, Renata Pinter, Dora Kolozsvari, Zsuzsanna Kovats, Andriy Yabluchanskiy, Cameron D. Owens, Zoltan Ungvari, Stefano Tarantini, Gabor Horvath, Veronika Muller, Janos Tamas Varga

**Affiliations:** 1 Department of Public Health, Faculty of Medicine, Semmelweis University, Budapest, Hungary; 2 International Training Program in Geroscience, Doctoral School of Basic and Translational Medicine/Department of Public Health, Semmelweis University, Budapest, Hungary; 3 Oklahoma Center for Geroscience and Healthy Brain Aging, University of Oklahoma Health Sciences Center, Oklahoma City, OK USA; 4 Coordination Center for Research in Social Sciences, Faculty of Economics and Business, University of Debrecen, Debrecen, Hungary; 5 Department of Pulmonology, Semmelweis University, Budapest, Hungary; 6 Doctoral School of Psychology, ELTE Eötvös Loránd University, Budapest, Hungary; 7 Institute of Psychology, ELTE Eötvös Loránd University, Budapest, Hungary; 8 Vascular Cognitive Impairment, Neurodegeneration and Healthy Brain Aging Program, Department of Neurosurgery, University of Oklahoma Health Sciences Center, Oklahoma City, OK USA; 9 Stephenson Cancer Center, University of Oklahoma, Oklahoma City, OK USA; 10 Department of Health Promotion Sciences, College of Public Health, University of Oklahoma Health Sciences Center, Oklahoma City, OK USA

**Keywords:** Long COVID, Post-COVID, Pulmonary rehabilitation, Quality of life, Functional parameters

## Abstract

The presence of prolonged symptoms after COVID infection worsens the workability and quality of life. 200 adults with long COVID syndrome were enrolled after medical, physical, and mental screening, and were divided into two groups based on their performance. The intervention group (*n* = 100) received supervised rehabilitation at Department of Pulmonology, Semmelweis University with the registration number 160/2021 between 01/APR/2021–31/DEC/2022, while an age-matched control group (*n* = 100) received a single check-up. To evaluate the long-term effects of the rehabilitation, the intervention group was involved in a 2- and 3-month follow-up, carrying out cardiopulmonary exercise test. Our study contributes understanding long COVID rehabilitation, emphasizing the potential benefits of structured cardiopulmonary rehabilitation in enhancing patient outcomes and well-being. Significant difference was found between intervention group and control group at baseline visit in pulmonary parameters, as forced vital capacity, forced expiratory volume, forced expiratory volume, transfer factor for carbon monoxide, transfer coefficient for carbon monoxide, and oxygen saturation (all *p* < 0.05). Our follow-up study proved that a 2-week long, patient-centered pulmonary rehabilitation program has a positive long-term effect on people with symptomatic long COVID syndrome. Our data showed significant improvement between two and three months in maximal oxygen consumption (*p* < 0.05). Multidisciplinary, individualized approach may be a key element of a successful cardiopulmonary rehabilitation in long COVID conditions, which improves workload, quality of life, respiratory function, and status of patients with long COVID syndrome.

## Introduction

Following the global pandemic and multiple waves of SARS-CoV-2 infections totaling 774 million confirmed cases, the world is now confronted with another challenge: the long COVID syndrome [1–3]. The long COVID syndrome is characterized by symptoms persisting beyond 12 weeks from the initial COVID-19 infection, without any alternative diagnosis to account for them [4, 5]. The significance of long COVID in older adults is particularly profound [2, 6], given the unique vulnerabilities of this demographic to both the acute and prolonged impacts of COVID-19 [7–11]. Older individuals, often grappling with pre-existing health conditions and diminished physiological resilience [8, 9, 12], are at heightened risk of experiencing the severe and enduring symptoms associated with long COVID [2, 13]. Supportive care is crucial in the initial 4 to 12 weeks following acute COVID-19 infection [14, 15]. The Centers for Disease Control and Prevention (CDC) classifies the long COVID condition as the presence of symptoms that emerge four weeks after the acute phase of COVID-19 infection [16]. Emphasizing the importance of the long COVID syndrome with the acknowledgment of the CDC definition and highlighting that even after 4 weeks of recovering from COVID-19 infection the presence of long COVID syndrome should be taken into consideration. Early recognition and involvement in rehabilitation significantly reduces the burden of the disease and improves the patient's quality of life. The CDC definition alerts alarming symptoms even after 4 weeks, aiding the healthcare provider in initiating the diagnostic process and establishing the primary diagnosis in a short time. However, World Health Organization (WHO) defines long COVID syndrome as a continuation or development of new symptoms 3 months after the SARS-CoV-2 infection, lasting for at least 2 months with no other explanation [17]. The impact of long COVID syndrome is growing, with an estimated global prevalence of approximately 43%, predominantly affecting the working, middle-aged population [18]. Long COVID syndrome impairs physical and cognitive functions, reducing resilience, societal participation, and overall quality of life, potentially leading to an inability to work [19–21]. Given its prevalence in the working-age group, long COVID also poses an economic burden [22–24]. Long COVID is associated with a range of general symptoms, including fatigue, malaise, attention disorders, and more specific conditions affecting several organs [6, 25, 26]. Respiratory and cardiovascular symptoms are among the most common, encompassing shortness of breath, cough, dyspnea, chest pain or tightness, fatigue, palpitations, arrhythmias, tachycardia, and exercise intolerance [6, 27, 28]. Notably, 20% of long COVID patients continue to experience prominent respiratory symptoms like shortness of breath and cough [29–31]. Additionally, long COVID can manifest in neurological, psychological, and gastrointestinal disorders, with symptoms such as imbalance, cognitive impairments, somatization, anxiety, depression, and loss of appetite [5, 32, 33]. These multifaceted manifestations highlight the complex and pervasive nature of long COVID syndrome, underscoring the need for comprehensive management strategies.

The COVID-19 pandemic remains a significant challenge for healthcare professionals globally [34–36], highlighting the ongoing need for rehabilitation programs to address the extensive pulmonary and neurological damage, multiple organ failure, and muscle damage caused by COVID-19 infections [37, 38]. The importance of complex cardiopulmonary rehabilitation as a key therapeutic intervention for COVID-19 survivors is increasingly recognized [39–42]. The emergence of long COVID necessitates urgent rehabilitation efforts [43–45]. The World Health Organization (WHO) recommends a person-centered, comprehensive, and multidisciplinary rehabilitation approach for individuals with long COVID conditions [46]. This approach may encompass physical activities, breathing techniques, exercise therapy, as well as social, psychological, and cognitive interventions, reflecting the holistic nature of effective rehabilitation programs [4, 15, 47].

Mitochondrial function plays a crucial role in cellular energy production, calcium homeostasis, and apoptosis regulation. Dysfunction in mitochondria can have various consequences on cellular functions, including immune response and viral infections [48]. Regarding COVID-19, there is emerging evidence suggesting that mitochondrial dysfunction might contribute to disease severity and complications [49]. Mitochondria are central to immune cell function, including the activation of immune responses [50]. Mitochondrial disfunction can impair the ability of immune cells to respond effectively to viral infections, including COVID-19 infection. This dysfunction can lead to a prolonged or dysregulated immune response, contributing to tissue damage and inflammation seen in severe COVID-19 cases. While the SARS-CoV-2 virus primarily replicates in the host cell's cytoplasm, it can still interact with mitochondrial components, potentially affecting mitochondrial function. Some studies suggest that mitochondrial dysfunction may facilitate viral replication, although the exact mechanisms in the context of COVID-19 infection are still under investigation. Mitochondrial dysfunction has been linked to the reactivation of latent viruses. In the context of COVID-19, there have been reports of viral reactivation, although the extent to which mitochondrial dysfunction contributes to this phenomenon requires further study [6, 51, 52].

Clinical trials have demonstrated the positive impacts of rehabilitation on the physical and cognitive functions of the human body [53]. Professional rehabilitation programs have been shown to enhance aerobic capacity, pulmonary function, muscle strength, muscle mass, physical performance, and subsequently, quality of life, cognitive functioning, and mental health [54]. Respiratory rehabilitation programs specifically target the improvement of spirometry parameters, exercise capacity, and quality of life through the strengthening of peripheral and respiratory muscles [55]. Regular breathing exercises, stretching, and resistance training have been found to improve diaphragm movement, exercise capacity, and maximal respiratory pressure [56]. Moreover, preoperative pulmonary rehabilitation, including controlled breathing techniques, stretching, resistance training, and personalized training, has significantly improved exercise capacity, chest expansion, quality of life, and lung mechanics [57, 58].

This study aims to validate the hypothesis that a tailored, ongoing cardiopulmonary rehabilitation program for patients with long COVID syndrome leads to increased vital capacity, improved general health condition, enhanced exercise physiological parameters, symptom relief, and predicts a better quality of life. To evaluate these hypotheses, patients with moderate to severe long COVID symptoms were enrolled and assessed through physical activity performance, breathing exercises, and questionnaires. The study employs non-invasive cardiopulmonary exercise testing (CPET) to monitor the effects of physical training, measuring exercise capability and identifying submaximal and peak exercise physiological responses [59–61]. CPET provides valuable insights into functional capacity, impairments, and exercise-related pulmonary and cardiac functional parameters, and is particularly useful for evaluating the severity of exertional dyspnea, exercise intolerance, or exercise-induced hypoxemia [62, 63].

## Methods

### Study group

**Fig. 1:**
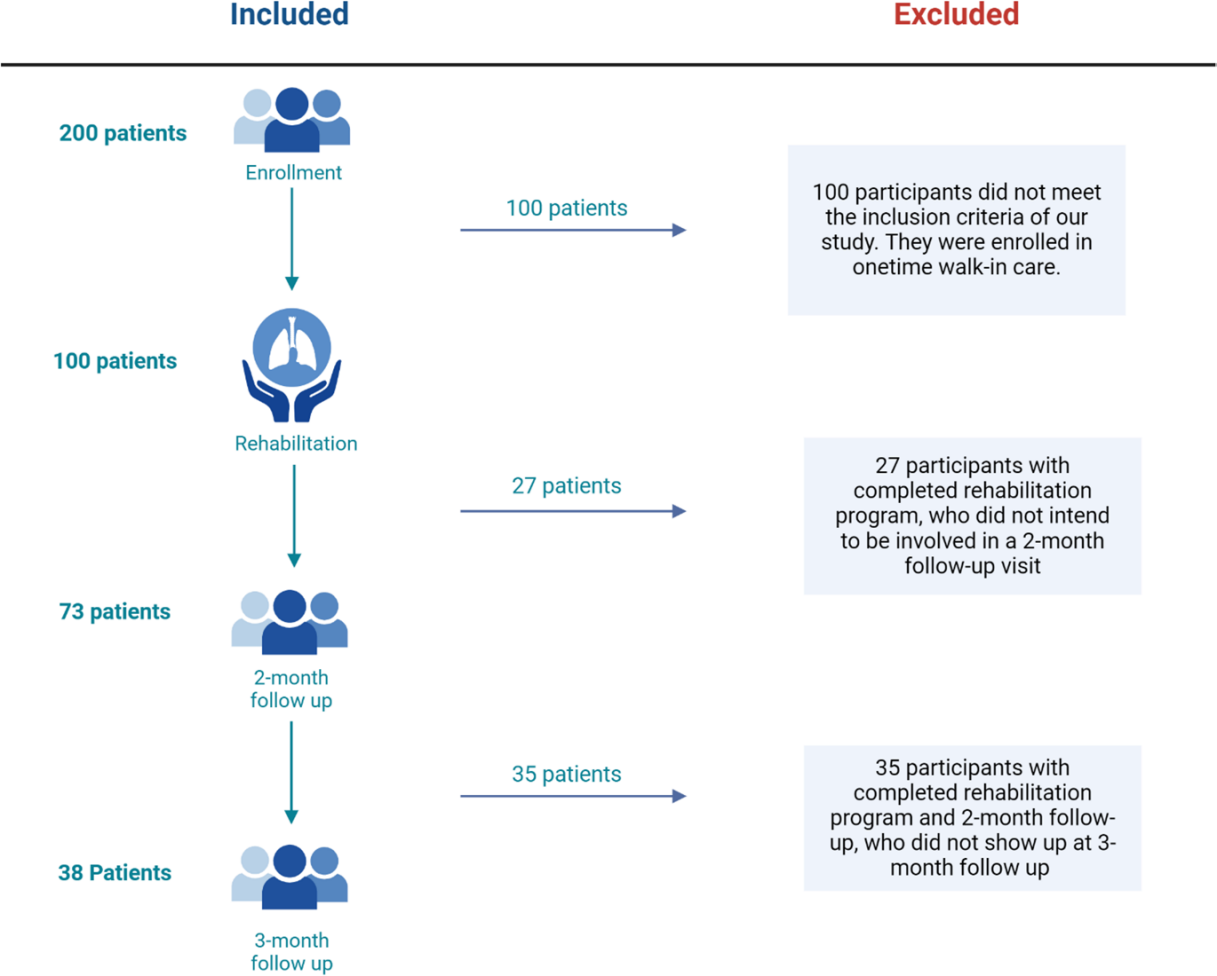
Study flowchart

One hundred middle aged adults, who recovered from COVID-19 and had durable long COVID symptoms, were recruited. An observational quasi-experimental study was conducted at the Department of Pulmonology, Semmelweis University. All procedures and study protocol were approved by the Ethical Committee of Semmelweis University with the registration number 160/2021, and it complies with the Helsinki Declaration. During the enrollment phase, all the potential participants were tested by Polymerase Chain Reaction (PCR) tests as a considered gold standard for COVID-19 infection diagnosis. Performing a negative PCR COVID-19 test involved collecting a sample from the nasopharynx (nasopharyngeal swab) and from the tonsils and the back of the throat (oropharyngeal swab) and analyzing the presence of the virus’s genetic material (RNA) [64, 65]. Presenting a negative PCR test was crucial, to exclude the possibility of viral reactivation caused by mitochondrial dysfunction, influencing oxygen uptake, and enrolling participants with real long COVID syndrome to assist the effectiveness of the cardiopulmonary rehabilitation program in this term [66]. Before assessment an oral and written informed consent was provided, enrollment started with a signed informed consent. The medical history, physical and mental status of the participants were screened to determine eligibility to participation. All participants were enrolled by their pulmonologist from the outpatient clinic of the Department of Pulmonology. During the enrollment process 200 people were culled (Fig. 1). According to their performance 100 age-matched patients (56.7 ± 12 years of age, 43 female and 57 male) received a one-time checkup with a draft of a home-based program without supervision and 100 participants (56.7 ± 14.2 years of age, 43 female and 57 male) were involved in the rehabilitation program. We compared the two groups’ performance, based on their pulmonary functional parameters, symptoms, quality of life related to their six-minute walking distance. The presentation of the comparison between the two groups, affected by different severity of long COVID syndrome, aims to provide a better understanding of the parameters that negatively influence everyday life in individuals with long COVID syndrome. In addition to the measured parameters, the clinical picture also had a huge impact on the eligibility for the rehabilitation program. Our hypothesis is that all these parameters can improve as an effectiveness of the rehabilitation program.

Machine learning determined an optimal classification model in the form of the Random Forest, a decision tree, to find reliable predictor for rehabilitation according to their performance [67]. The importance of each feature is assessed during the training process, and the collective importance across all trees helps identify the most influential features [68]. Our main outcome for the Random Forest was respiratory functional parameters, six-minute walking test (6MWT) and quality of life.

**Fig. 2:**
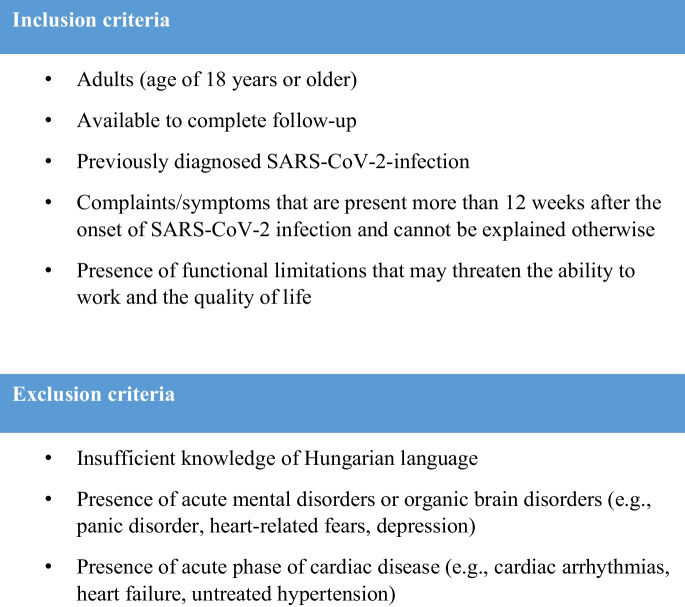
The eligibility criteria and exclusion criteria for long COVID rehabilitation

Inclusion criteria for the rehabilitation program consisted of previous diagnose and/or hospitalization with SARS-CoV-2-infection and durable long COVID symptoms (≥ 12 weeks after onset of the acute SARS-CoV-2 infection and cannot be explained otherwise), the presence of functional limitations that may threaten the everyday life and ability to work and understand oral and written Hungarian language. Individuals with any of the following conditions were excluded from our study: acute mental disorders or organic brain disorders, uncontrolled hypertension (≥ 140–90 mmHg), uncontrolled diabetes mellitus, unstable cerebrovascular or significant cardiac disease (e.g., heart failure, angina NYHA Class III-IV), severe rheumatic or orthopedic condition or any acute, urgent and/or potentially life-threatening clinical condition (Fig. 2.) As part of the prior medical screening, patients underwent numerous medical examinations according to the recommendation of CDC requirement of Assessment and Testing for Post-COVID Conditions [16]. Preliminary examination screened complete physical (including cardiovascular examination), emotional, and behavioral status. The focus was on the comorbidities, because multi-comorbidities especially worsened the severity of COVID-19 infection and made patients require rehabilitation in the future [69]. The most common comorbidities among our study population were cardiovascular disease (70%), respiratory disease (30%) [70]. Vaccination status was not taken into consideration, as in Hungary, the availability of vaccines was extremely limited at the first quarter of 2021. Only certain groups received vaccinations in the first quarter due to the limited supply of vaccines. Therefore, a significant percentage of patients who entered the rehabilitation program until late 2021 were unvaccinated, as enrolment took at least 3 months, based on the WHO long COVID definition, after the actual recovery from COVID-19 infection. Mass vaccinations for the working-age population became available at the end of March 2021. However, vaccine hesitancy hindered vaccination coverage. In July 2021, a law was passed on mandatory vaccination. The rehabilitation program, along with the start of our research, began during a transitional period with a shortage of vaccinations. Due to the initial difficulties with vaccination, we decided not to take vaccination status into account [71–74]. After excluding all the urgent and potentially life-threatening conditions (coagulation disorders, myocardial infarction, stroke, renal failure) [16], baseline measurements were carried out. The same measurements were repeated after the 14-day rehabilitation program, the findings were published in our earlier work [75]. To validate the long-term positive effect of the 2-week long rehabilitation on long COVID participants CPET was carried out at the 2- and 3-month follow-up. The associations between cardiopulmonary rehabilitation, patients’ performance, and the impact on their quality of life were also analyzed.

### Study design

The rehabilitation was designed according to the newly accepted long COVID rehabilitation protocol [39], supervised, and run by cardiopulmonary rehabilitation specialists and their team in the pulmonology department. The team that was responsible for the 2-week rehabilitation contained a pulmonologist, cardiologist, physiotherapist, dietitian, psychologist, and social worker. The person-centered rehabilitation program included individual and group sessions, e.g., individualized low to moderate intensity exercise training, breathing exercise training as well as dietary and social counselling. In our study, only low-intensity exercise training was involved, considering our patients’ safety. This decision was based on previous research, that found interval training and graded exercises can be harmful for long COVID patients [76]. The association between the severity of SARS-CoV-2 infection and a higher risk for major cardiovascular events has been proved [77].

Our research group considers a personalized, effective rehabilitation program through outpatient rehabilitation in small groups of 6–8 participants every time biweekly, which may be a more beneficial form of patient care. Patients usually spend their time at the clinic from 8 AM to 4 PM, while individual and group sessions take place. Due to this form of care, participants in rehabilitation do not require hospitalization. Because of the small group size, the rehabilitation specialist group can pay special attention to ensuring that patients fully understand and properly execute the tasks aimed at long-term improvement, enabling them to perform safely and independently at home. Before completing the program, healthcare professionals involved in rehabilitation ensure that patients understand and correctly perform the tasks. Sustainable maintenance programs that can be integrated into the clinic are needed, which may include telephone or online counseling, and personal meetings if necessary. Considering the positive outcomes of telerehabilitation in different fields of rehabilitation, tailored to the specific patient group, it could be reasonable to become part of the long-term follow-up in long COVID rehabilitation as well [78].

### Interventions

Rehabilitation program included a 30-min group breathing exercise session, 3 sessions/day, led by a professional physiotherapist (controlled breathing technique, chest mobility-enhancing and muscle-strengthening exercises with own body weights and dumbbells as well), and a 30-min individualized low-intensity continuous individual exercise training (arm ergometer, stationary bicycle, treadmill or rowing machine), 2 sessions/day, where severity of symptoms, age, comorbidities, and current conditions were taken into consideration [79, 80]. When designing individual exercise programs, we considered factors such as the severity of previous COVID-19 infection, possible comorbidities (e.g., pre-existing obstructive lung disease, high blood pressure, heart failure, pulmonary hypertension, respiratory insufficiency, joint diseases), general health condition and physical parameters. In severe cases, we implemented high-intensity interval training. In other cases, the intensity of exercise was kept at 60–80% of maximal intensity. During rehabilitation, under strict supervision and we gradually increased the duration of exercise, reaching a weekly maximal 15% increment. High-intensity interval training includes short periods of intense activity and periods of lower-intensity or rest. It typically involves pushing effort near to maximum during the high-intensity intervals, followed by brief recovery periods. During the 10-min warm-up phase of light aerobic exercise participants prepare for the workout. Performing exercise (such as walking, cycling) at maximum effort for a short 60-s duration. During the recovery interval they rest or perform a lower-intensity exercise for 60 s. An alternation was used between high-intensity intervals and recovery intervals for a predetermined number of cycles. Untrained participants with severe syndromes started with 4–6 cycles, while individuals with better health conditions may do 8 or more. At the end of a 10-min cool-down phase of light aerobic exercise brings down heart rate and prevents dizziness or lightheadedness. The benefit of high-intensity interval training is its efficiency. It allows patients with long COVID syndrome to improve cardiovascular fitness in a shorter amount of time compared to steady-state cardio exercises. Additionally, it has been shown to increase metabolism, improve insulin sensitivity, and enhance oxygen consumption post-exercise, leading to overall fitness improvements. However, it is important to start gradually and be supervised by a healthcare professional, especially in a study population with underlying health conditions [81–86].

**Fig. 3:**
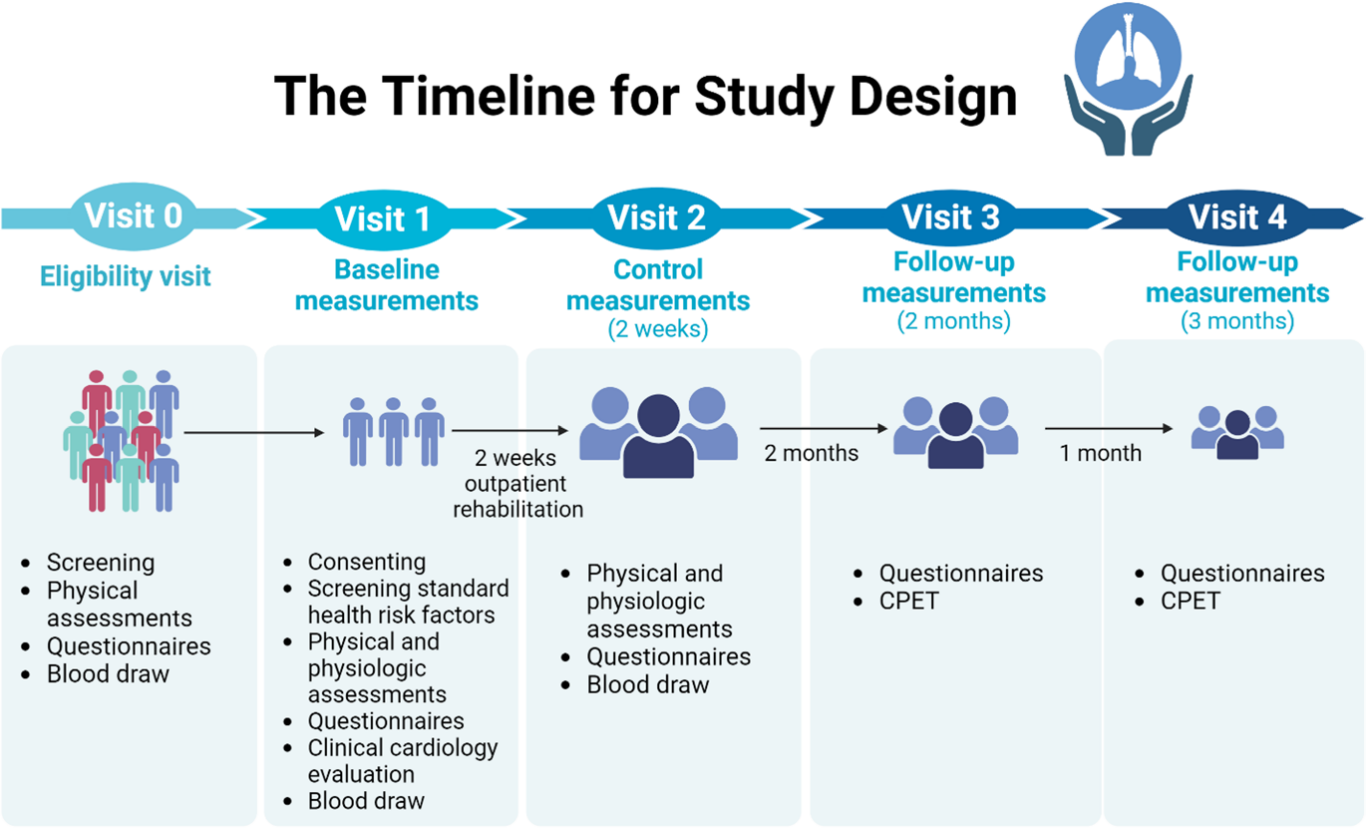
The timeline for study design

The present study aimed to monitor the long-term effects of cardiopulmonary rehabilitation, proving that a 2-week long program can increase vital capacity and provide better general health condition, a relief of symptoms and these alterations predict a better quality of life with a permanent, individualized cardiopulmonary rehabilitation program in patients with manifested long COVID syndromes. The clinical diagnosis of long COVID was based on the WHO definition of long COVID condition, as condition refers to symptoms that usually lasting 12-week from the onset of SARS-CoV-2 infection and cannot be explained by any alternative diagnosis [4]. Performance on physical activity, breathing exercise and questionnaires, among others were measured in patients with moderate or severe symptoms of long COVID condition and compared their baseline performance to the 2-week, showing significant improvements [75]. CPET was carried out at 2-month and 3-month follow-up measurements. The timeline for study design and investigative measurements is represented in Fig. 3. After the 2-week rehabilitation program, the participants did not get any additional treatment, but they were advised to continue practicing the learned exercises at home daily. One of the cornerstones of the 2-week rehabilitation program is the continuous supervision, ensuring that patients understand and correctly master the tasks to be performed at home. Additionally, they receive written materials for home use. Furthermore, at the end of the rehabilitation program, vital signs are reassessed, confirming their stability, and thus reducing the risk level. In case patients experience any negative changes (e.g. difficulty breathing, chest pain), immediate consultation with the pulmonology rehabilitation specialist involved in the program is possible. Seventy-three participants out of 100, who successfully completed the rehabilitation program, showed up at 2-month follow-up; however, only 38 patients participated in the 3-month follow-up. However, the dropout rate during the follow-up phase seems to be high, it is important to highlight that the 2-week rehabilitation program was successfully completed by all the enrolled participants. The higher dropout rate in the follow-up phase can be explained by the compliance decline as participants who completed the rehabilitation were released with advice but not directly involved in further supervised or controlled maintenance program. They were asked to come back for a 2- and 3-month follow-up, but not as part of their rehabilitation program. The dropout in the rehabilitation program itself was 0%, but the patient compliance in the follow-up phase can occur for various reasons, such as personal reasons. With a successfully completed rehabilitation program, participants recovered and gained back their workability and quality of life. It could lead to significantly decreased compliance and loss of interest, as they no longer expect any further benefit from the program. The patient non-compliance rate in follow-up phase can vary significantly depending on length of the study, the nature of the intervention or treatment being studied, participant characteristics, and study design. Compliance significantly declines, especially in studies involving long-term follow-up or interventions with demanding requirements [87–89].

Our second aim was to create a model which assists during the enrollment process by assigning feature importance scores to variables (such as symptoms, physical parameters) to create an optimal classification model for people with long COVID syndrome. Random Forest was picked as a possible machine learning methos, because of its ability of handling mixed feature types and low-dimensional data. The subsequent section will define and discuss the obtained results in detail.

### Safety consideration

The rehabilitation program was considered as minimal health risk and associated with routine medical examinations and testing. However, there were rare potential risks involved in some procedures. During CPET (electronically braked cycle ergometer and the low-intensity continuous exercise training), there was a possibility of shortness of breath, fatigue, chest pain, if the participant had severe pulmonary long COVID symptoms and a higher risk for major cardiovascular events. Therefore, all participants underwent a strict, extensive medical examination and questionnaire before the rehabilitation program. Blood pressure and oxygen saturation were monitored by a personal physiotherapist during the whole session. If history of COPD or chronic cardiovascular disorder (e.g., coronary heart disease, stroke, peripheral arterial disease, or aortic disease) was presented, the participant could be eligible for less demanding exercise.

## Measurements

### Spirometry

Automated computerized spirometer was used to monitor respiratory parameters. Body plethysmography defined dynamic lung volumes, e.g., forced expiratory volume in 1 s (FEV1%pred), forced vital capacity (FVC %pred), the degree of airway obstruction (FEV1/FVC) with Global Lung Function Initiative-defined (GLI-defined) normal spirometry [90]. Spirometry measures the air volume and flow during inhalation and exhalation. Forced Vital Capacity (FVC) is the maximum air volume that can be forcefully exhaled after a deep breath. It represents the total lung capacity and is used to assess the overall lung function. Forced Expiratory Volume in 1 s (FEV1) is the air volume forcefully exhaled in the first second. It indicates the rate at which air can be forcibly expelled from the lungs and is a measure of airflow limitation. FEV1/FVC ratio shows the value of the FVC that is exhaled in the first second [91, 92]. Diffusion capacity measurements were also performed.

### Lung diffusion test

Lung diffusion test measures carbon monoxide uptake in the lung. It measures how well gases, particularly oxygen and carbon monoxide, move from the lungs into the bloodstream. This test assesses the efficiency of gas exchange in the lungs, specifically the ability of the lungs to transfer oxygen from the air into the bloodstream and remove carbon dioxide. The results of the test help healthcare providers assess the severity of lung diseases, determine treatment effectiveness, and guide decisions regarding pulmonary rehabilitation or supplemental oxygen therapy [93]. It can be described by the transfer factor for carbon monoxide (TLCO) or diffusing capacity for carbon monoxide (DLCO). The transfer coefficient for carbon monoxide (KLCO) was also calculated [94].

### Oxygen saturation

During the individualized training, the pulse and the oxygen saturation were detected with a noninvasive pulse oximetry constantly to monitor the physiological changes and avoid harmful effects and stop the training if it is needed [95].

### The 6-min walk test (6MWT)

As cardiopulmonary functional test 6MWT detects the degree of functional impairment. 6MWT is carried out by walking the aisle with marked turnaround points for 6 min with pulse oximeter on. 6MWT measures the covered distance (m) walked unassisted on a hard and flat surface in 6 min [96].

### Euro quality of life—5-dimension questionnaire—3-level (EQ-5D-3L)

The EQ-5D is a widely used, reliable questionnaire that measures health-related quality of life in five dimensions: mobility, self-care, usual activities, pain/discomfort, and anxiety/depression. EQ-5D points how rehabilitation affects the generic quality of life with three possible answers for each item (1: no problem, 2: moderate problem, 3: severe problem) [97].

### Cardiopulmonary exercise testing (CPET)

Cardiopulmonary exercise testing (CPET) with maximal exercise ability measurement was performed under the direction of a pulmonary specialist. Our study included an electronically braked cycle ergo spirometer (Ergoline-900, Piston Medical, Budapest, Hungary). Cardiopulmonary exercise testing is commonly used for the evaluation of exercise intolerance and exercise-related symptoms and exercise responses involving the pulmonary and cardiovascular system [98]. Cardiopulmonary exercise testing provides reliable data on patients’ current health conditions and shows the changes during the given period [99]. During the whole measurement, a real-time electrocardiogram (ECG) was detected for patients’ safety [62]. CPET provides direct noninvasive information about minute ventilation, heart rate, oxygen consumption and carbon dioxide production at rest and during exercise and pulse oximeter saturations [100].

### Pulmonary function and exercise test

As part of the measurement patients performed pulmonary function tests supervised by specialists including spirometry, body plethysmography and diffusion capacity at 2- and 3-month control visit. After 10-min resting phase and 3-min constant pedaling at 20 W, then work rate was increased 5, 10, or 15 W/min in ramp profile. The pedaling rate was kept at approximately 60 rpm constantly. The ergometer was calibrated before each test. During the whole measurement gas analysis, forced expiration and inspiration capacity measurement (e.g., Forced Vital Capacity (FVC), Forced Expiratory Volume in 1.0 s (FEV1) or Peak Expiratory Flow (PEF)), static vital capacity measurement (e.g., Inspiratory Vital Capacity (IVC)) and Maximal Voluntary Ventilation (MVV) measurement, and heart rate, ECG recording and oxygen saturation by pulse oximetry was carries out [98].

### Statistical analysis

All statistical analyses were conducted with STATA 14 (StataCorp, College Station, TX). Most of our continuous data did not follow normal distribution, verified by Shapiro–Wilk test and non-parametric statistical methods were used. Continuous variables were shown by medians and interquartile ranges. Categorical data were presented with case numbers and proportions. To analyze the differences of continuous variables between the two groups, we used Mann–Whitney test. Frequency differences of categorical variables were tested by Fisher’s exact test. The significance limit used was *p* < 0.05. Random Forest was trained on the 200 individuals’ dataset, to predict the outcome. Based on the collected data, the system determined whether the subject belonged to the rehabilitation or outpatient category, aiming to establish a reliable and interpretable classification model for long COVID rehabilitation. The chosen algorithm (BMI, FVC, FEV1, FEV1/FVC, PEmax, PImax, Sp02, 6MWT, EQ-5D) underwent ten runs to ensure a comprehensive assessment of its performance. In Random Forest classification, accuracy is typically calculated as the percentage of correctly classified instances out of the total number of instances in the dataset. Accuracy is a commonly used method to evaluate the performance of a classification model. The median of each accuracy results showed in mean accuracy indicating as a reliable predictor for a possible need of rehabilitation. We showed that functional parameters, oxygen saturation, and quality of life were decreased in long COVID participants compared to outpatient clinic group. oxygen saturation and functional parameters (PImax, FEV1) had the highest feature importance in Random Forest group classification. The selected parameters and their importance collectively affirm the reliability and generalization capability of the developed classification model [68].

## Results

During the enrollment phase differences in various respiratory functional parameters between the two groups (outpatient group [OPC] vs. intervention group with 2-week rehabilitation [PR]) were measured. This characteristic was found to be statistically significant, highlighting the distinct physiological characteristics associated with each group at the commencement of the study. In restrictive ventilatory defects, both lung and chest compliance decrease, consequently reducing static lung volumes. In long COVID condition, shown in our study, FVC and FEV1 values decrease, since the airways are generally not affected by the conditions, and the FEV1/FVC ratio shows a slightly elevated value. If FVC/FEV1 ratio is higher than normal, it indicates a restrictive pattern [101, 102]. Significant difference was detected at FVC (L) 3.36 (2.7–4.1) vs. 3(2.36–3.57) *p* = 0.011, FEV_1_(L) 2.82 (2.27–3.38) vs. 2.51 (1.98–3.06) *p* = 0.006, and FEV_1_ (ref%) 92 (79.5–104) vs. 84.88 (75.15–89.19) *p* < 0.001 and SpO_2_ (%) 98 (97–99) vs. 96 (95–98), *p* < 0.001 (Table 1).

**Table 1:** Anthropometric and functional data of patients participating in outpatient clinic (n = 100) and participants enrolled in 2-week pulmonary rehabilitation program (n = 100)

Variables	Out-patient clinic (OPC) n = 100	Rehabilitation (PR) n = 100	*p*-value
Age (years) (IQR)	56 (48–68)	56 (47.8–66)	0.881
Male/Female (n, %)	57/43 (57%, 43%)	57/43 (57%, 43%)	N/A
BMI (kg/m^2^)	28.75 (25.22–33.11)	28.87 (26.48–33.49)	0.504
FVC (L)	3.36 (2.7–4.1)	3 (2.36–3.57)	0.011*
FVC (%)	86 (73.5–96)	86 (69.5–97.5)	0.585
FEV_1_ (L)	2.82 (2.27–3.38)	2.51 (1.98–3.06)	0.006*
FEV_1_ (ref%)	92 (79.5–104)	84.88 (75.15–89.19)	< 0.001*
FEV_1/_FVC (L)	83.13 (78.26–88.13)	84.57 (74.82–88.29)	0.593
FEV_1/_FVC (%)	106 (102–111)	107 (96.5–112)	0.627
TLCO (mmol/min/kPa)	5.45 (4.49–6.52)	6.87 (5.72–8.54)	< 0.001*
TLCO (%)	94.5 (84–110)	90 (74–108)	0.121
KLCO (L)	1.65 (1.41–2.01)	1.6 (1.38–1.9)	0.404
KLCO (%)	111 (88–131.5)	85.5 (75–107)	< 0.001*
DLCO (L)	8.86 (7.18–11.16)	10.68 (7.22–12.59)	0.190
DLCO (%)	111.5 (94.5–127)	108.5 (86–130)	0.462
PEmax (kPa)	8.84 (7.25–10.92)	9.8 (7.27–11.68)	0.550
PImax (kPa)	7.41 (5.31–9.86)	7.35 (5.01–10.8)	0.518
SpO_2_ (%)	98 (97–99)	96 (95–98)	< 0.001*
6MWT distance (m)	477 (402.5–502.5)	471 (368.5–534.5)	0.966

Data are presented as median (IQR); IQR = interquartile range; BMI = body mass index; FVC = forced vital capacity; FEV1 = forced expiratory volume in 1 s; FEV1/FVC = forced expiratory volume in the first one second to the forced vital capacity; TLCO = transfer factor for carbon monoxide; KLCO = transfer coefficient for carbon monoxide; DLCO = diffusing capacity for carbon monoxide; PEmax = maximal expiratory pressure; PImax = maximal inspiratory pressure; Spo_2_ = blood oxygen saturation; 6MWT = six-minute walk test; p < 0.05 means the two indicators were significantly correlated

**Fig. 4:**
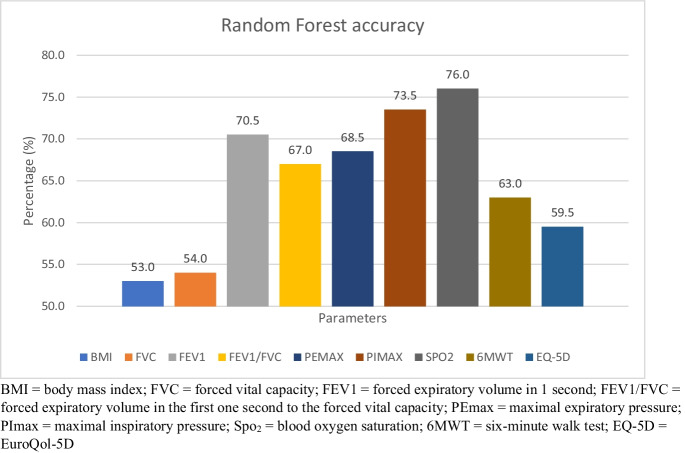
Random Forest, a Machine learning determined an optimal classification model to find reliable predictor for rehabilitation according to participants’ performance

Random Forest with 10 runs assigned importance scores to each feature having the highest importance value in SpO_2_ (76%), PImax (73.5%) followed by FEV1 (70.5%). The importance value of 6MWT was 63% and EQ-5D was 59.5%. Model evaluation benefits significantly from diverse datasets (pulmonary functional parameters, 6MWT and EQ-5D) and multiple test-train splits, enhancing robustness. Exploring various scenarios uncovers potential biases, providing a holistic view of the model’s generalization across contexts. Examining selected features and their importance offers insights into the model's functionality. Aggregated metrics provide a global assessment, while individual metrics offer nuanced insights into strengths and weaknesses in specific contexts of pulmonary functional parameters, 6MWT and quality of life (EQ-5D) in long COVID rehabilitation (Fig. 4).

**Fig. 5:**
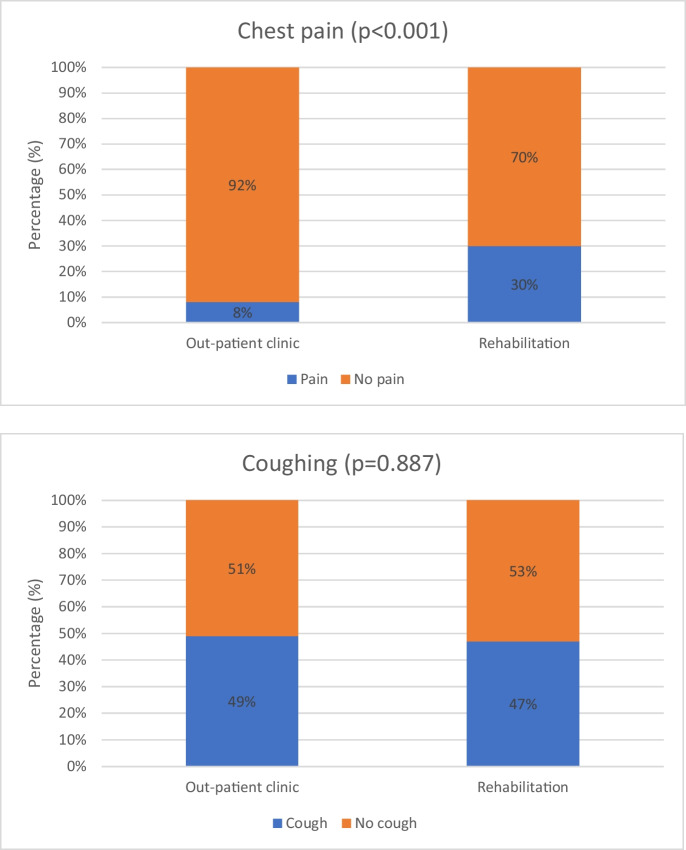
Main symptoms that were characteristic during the enrollment period, comparing the two group: out-patient clinic (*n* = 100) vs. participants enrolled in pulmonary rehabilitation (*n* = 100)

The most common subjective symptoms of long COVID patients included reduced performance, shortness of breath, productive cough, dyspnoea, chest pain (e.g. retrosternal pressure). Productive cough appaired as a common symptom in both group; in OPC group 49%, in PR group 47%, experienced regular coughing. Chest pain was more characteristic in PR group at the enrolment phase; 30% reported, while in OPC group only 8% (Fig. 5).

EQ-5D pointed out the main difference in quality of life between the two groups with significant difference in pain/discomfort dimension (OPC vs. PR, Mean (SD), 1.53 (0.59) vs. 1.72 (0.52), *p* = 0.009 (Table 2).

**Table 2:** Differences between EQ-5D-3L instruments in patients participateing in outpatient clinic (n = 100) and rehabilitation program (n = 100)

Variables	Out-patient clinic (OPC) n = 100	Pulmonary rehabilitation (PR) n = 100	*p*-value
EQ-5D mobility	1.39 (0.51)	1.42 (0.5)	0.567
EQ-5D self-care	1.15 (0.41)	1.1 (0.3)	0.479
EQ-5D usual activities	1.56 (0.61)	1.6 (0.62)	0.691
EQ-5D pain/discomfort	1.53 (0.59)	1.72 (0.52)	0.009*
EQ-5D anxiety/depression	1.43 (0.57)	1.49 (0.58)	0.405

EQ-5D-3L describes quality of life in 5 dimensions (mobility, self-care, usual activities, pain/discomfort, anxiety/depression). Scores represent 1: no problem, 2: moderate problems, 3: extreme problems. Data are presented as mean ± SD. Significance was accepted at p < 0.05

Our study proved that 2-week cardiopulmonary rehabilitation permanently improves the participants’ performance. Our CPET data showed increased performance at the 3-month follow-up compared to the 2-month follow-up. Significant differences were detected in performance of VO_2_max (ml/min) 1276 (1070–1614)vs. 1429.5(1191–1871) *p* = 0.033, VO_2_/kg (ml/min/kg) 14.6 (11.95–18.2) vs.16 (12.4–21)*p* = 0.021 (Table 3).

**Table 3:** The CPET result of 2-month follow-up compared to 3-month follow-up

Variables	CPET (2-month)	CPET (3-month)	*p*-value
Peak work rate (WR)	106 (82—139)	121.5 (89—151)	0.084
HR (1/min)	135 (119—155)	141 (120—160)	0.948
HRR (1/min)	29 (11—51)	26.5 (11—43)	0.918
VO_2_max (ml/min)	1276 (1070—1614)	1429.5 (1191—1871)	0.033*
VO_2_/kg (ml/min/kg)	14.6 (11.95—18.2)	16 (12.4—21)	0.021*
VCO_2_max (ml/min)	1458 (1212—2021)	1696.5 (1363—2223)	0.129
VE max (L/min)	53.2 (43.3—67.5)	56.05 (45.5—79.3)	0.052
VE/VO_2_max	40.8 (35.9—47.2)	40 (35.9—44.5)	0.531
VE/VCO_2_max	38.4 (34.6—49.2)	40.7 (37.3—46.5)	0.291

Data are presented as median (IQR). IQR = interquartile range; HR = heart rate; HRR = heart rate reserve; VO_2_max = maximal oxygen consumption; VO_2_/kg = oxygen used in one minute per kilogram of body weight; VCO_2_ = carbon dioxide production; VEmax = Maximal ventilation; VE/VO_2_ = minute ventilation divided by oxygen production; VE/VCO_2_ = minute ventilation divided by carbon dioxide production; p < 0.05 means the two indicators were significantly correlated

No adverse event was recorded related to our rehabilitation program.

## Discussion

Our study’s findings reveal distinct differences between two groups (OPC vs. PR), indicating that the assessed parameters could significantly predict the need for rehabilitation in long COVID cases. Notably, disparities were especially evident in the pain/discomfort dimension of the EQ-5D-3L questionnaire. Considering the program’s focus on the working-age population, the emphasis on shorter yet effective rehabilitation courses is crucial for facilitating a rapid return to the workforce. Despite the seeming brevity of a 2-week intervention, our research confirmed its lasting positive effects, observable even three months after rehabilitation. This underscores the program's effectiveness, significantly enhanced by cardiopulmonary exercise testing (CPET).

A fundamental aspect of our multidisciplinary program is empowering individuals to master and practice the rehabilitation exercises at home, thereby lessening both health and economic impacts. This approach fosters self-sufficiency, encouraging participants to continue their rehabilitation journey independently of clinical settings. The focus on home-based practice not only augments the program's efficacy but also significantly reduces the health care and economic burdens of extended rehabilitation periods.

Our comprehensive analysis demonstrated that the rehabilitation program not only accelerates the recovery process but also secures sustained positive outcomes for participants. Initial studies on complex respiratory rehabilitation for long COVID patients have documented improvements in lung function and quality of life, highlighting the benefits of a regimen that includes respiratory muscle training, cough exercises, and diaphragm stretching performed over 6 weeks [62].

The rehabilitation journey extends to patient education, emphasizing the importance of physical activity and rehabilitation’s benefits [103–106]. Many participants presented potential lung damage and other long COVID symptoms, such as cough, shortness of breath, chest pain, headache, muscle pain, weakness, thromboembolic complications, and metabolic disturbances [5, 107–109]. The protocol of our pulmonary rehabilitation program focused on easing symptoms and improving quality of life by strengthening the respiratory muscles and auxiliary muscles and teaching breathing exercises and increasing exercise tolerance by regular supervised continuous exercise training [110–112].

Recent research corroborates the medium- to long-term efficacy and safety of pulmonary rehabilitation for moderate to severe long COVID cases, with significant gains in exercise capacity and pulmonary function reported after six weeks [111, 113, 114]. Some recent studies also provided first-hand experiences in connection to multidisciplinary long COVID rehabilitation, which contained different kind of physical training, just like aerobic training, resistance training, and breathing exercises [115–118]. However, despite these promising findings, gaps remain in our understanding of the optimal protocols, durations, and the cost-effectiveness of rehabilitation efforts. Our study contributes to this knowledge base by monitoring the progression and impact of physical exercises, offering insights into their safety, adherence, and benefits for long COVID recovery [119].

Decreased patient compliance is a leading problem after a successfully completed rehabilitation program. Patients return to the workforce, leading to a significant decrease in patient participation willingness, as also indicated by the statistics. Sustaining participation in the program would require additional effort, including more technical and personal resources, including regular follow-up calls. However, it is important to note that these participants have been absent from work significantly due to their COVID-19 infection, hospitalization and the prolonged recovery period burdened by long COVID syndrome, making it challenging to recall them for regular check-ups and measurements on workdays. We truly believe that long-term follow-up is necessary, ideally at 6 months and 1-year post-rehabilitation. However, our current resources are insufficient for this, especially considering the significantly reduced participation willingness [120, 121].

To assess the impact of physical training on long COVID patients, non-invasive cardiopulmonary exercise testing (CPET) was employed. This method served not only to measure exercise capacity but also to identify both submaximal and peak exercise responses [59, 122, 123]. CPET provided an objective evaluation of exercise tolerance, offering insights into the patients' functional capacity and any impairments, as well as specific exercise-related pulmonary and cardiac functional parameters [62, 124]. Furthermore, CPET is extensively recognized for its utility in diagnosing the severity of exertional dyspnea, exercise intolerance, or exercise-induced hypoxemia, making it an invaluable tool in the comprehensive evaluation of long COVID rehabilitation outcomes [63, 125, 126].

Patients with long COVID experienced a decline in functional capacity, leading to deteriorated quality of life and overall health [127–131]. Self-reported health questionnaires administered months after COVID-19 infection revealed that patients continued to suffer from persistent symptoms, reporting lower life satisfaction and poorer health outcomes, without significant differences between genders [132–136]. This aligns with our findings; patients noted a decline in their quality of life and general health upon enrollment, which significantly improved following rehabilitation.

Research indicates that the impact of long COVID can be exacerbated by the initial severity of the SARS-CoV-2 infection, age, and psychological stressors [6, 137]. The role of a multidisciplinary rehabilitation team is crucial in addressing these challenges, including mitigating fears associated with the condition and alleviating feelings of isolation, thereby enhancing overall health and quality of life [138–143]. Our team, comprising various specialists, is dedicated to supporting the comprehensive recovery of patients with long COVID. This multifaceted approach has demonstrated marked improvements in patients’ general health and quality of life, with notable progress observed just 2 weeks into the outpatient rehabilitation program, and continued improvement seen at 2- and 3-month post-rehabilitation.

Various rehabilitation programs have demonstrated significant enhancements in physical functioning, such as improvements noted in the 6MWT, for patients with long COVID [41, 113, 144–147]. Specifically, cardiopulmonary rehabilitation programs incorporating low-intensity exercise training have been successful in aiding the recovery of COVID-19 survivors, leading to an uptick in daily physical activities [42, 148–150]. Evidence underscores the necessity of both functional rehabilitation and physical support to ameliorate the condition of patients [150–152]. While some studies suggest that men might experience greater improvements in physical functioning [153–155], our findings did not reveal any gender disparities [156]. We observed that controlled, supervised outpatient rehabilitation programs offer long-term benefits to patients with long COVID, with significant enhancements in both health condition and physical functioning as verified by cardiopulmonary exercise testing (CPET) assessments.

Research exploring the impact of respiratory muscle training on exercise tolerance found that programs focusing solely on respiratory muscle training were less effective compared to more comprehensive rehabilitation strategies [157–160]. This is attributed to the fact that respiratory muscle training does not dynamically engage the cardiovascular system, which is crucial for patients experiencing long COVID symptoms [161]. Multidimensional exercise training, encompassing various modalities, was found to be more beneficial in improving both inspiratory and expiratory muscle strength and endurance [162–164]. Furthermore, personalized training programs were identified as particularly effective [165–167]. Given the heightened risk of major cardiovascular events among long COVID patients [168–171], our study opted for low-intensity training regimens to prioritize safety. Despite the lower intensity, these training programs incorporated a variety of exercise modalities designed to enhance physical condition and functionality, thereby improving the overall quality of life for individuals with symptomatic long COVID syndrome [172–174].

Another study, utilizing cardiopulmonary exercise tests on a cycle ergometer, revealed that a personalized rehabilitation program with a multicomponent training approach significantly enhanced respiratory muscle strength and muscle performance [175, 176]. This improvement led to a reduction in symptoms such as dyspnea and fatigue, fundamentally improving the physical condition and overall quality of life for participants [62, 177]. This finding challenges the notion that longer rehabilitation programs, extending six weeks or more, are inherently more effective for treating long COVID conditions [178, 179]. In a randomized controlled trial, no significant difference was observed in Forced Expiratory Volume in 1 s (FEV1), aligning with other research indicating that FEV1 typically does not change post-pulmonary rehabilitation in most respiratory pathologies [180, 181]. However, other metrics like Transfer Factor of the Lung for Carbon Monoxide (TLCO), Forced Vital Capacity (FVC), and levels of dyspnea showcased significant improvements from pre- to post-rehabilitation visits, without a clear correlation with the duration of rehabilitation [180]. These outcomes are consistent with studies demonstrating that a three-week pulmonary rehabilitation program can lead to improved FVC [75, 148, 182–184]. Our research, using CPET to track the extended effects of a 2-week rehabilitation program, found that this duration was optimal for inducing permanent change, evidenced by significant improvements in respiratory functioning at the 3-month mark.

## Conclusion

In summary, long COVID syndrome precipitates both physical and mental health decline. Yet, a tailored, consistent rehabilitation program has proven effective in aiding the recovery of physical and mental health in long COVID patients. For the working, middle-aged demographic, a shorter, yet impactful, rehabilitation program is crucial to minimize sick leave and facilitate a quicker return to employment and daily life. Our findings demonstrate that a 2-week intensive, patient-focused pulmonary rehabilitation program significantly boosts general health and aids participants in resuming their normal lives. This evidence underscores the necessity for cardiopulmonary rehabilitation programs that enhance both physical and mental health, thereby improving the quality of life for those affected by long COVID syndrome.

### Limitations

The study faced several challenges: initially, 100 patients enrolled in the 2-week rehabilitation program, but only 73 returned for the 2-month follow-up, and just 38 attended the 3-month assessment, indicating a dropout rate of 27% at 2 months and 62% at 3 months in the follow-up phase. Secondly, the original study design did not include arrival CPET at the beginning of the rehabilitation program. Additionally, the study lacked comprehensive data on COVID vaccination statuses, although this was not deemed crucial to evaluating the rehabilitation's impact. Lastly, the pre-enrollment physical examination was designed to screen for post-exertional symptom exacerbation (PESE) and post-exertional malaise (PEM), opting against the use of the DePaul Post-Exertional Malaise Questionnaire for enhanced patient safety.

## Data Availability

The data that support the findings of this study are available on request from the corresponding author (JTV).
